# Characterization of a novel model for chronic migraine

**DOI:** 10.1186/1129-2377-14-S1-P81

**Published:** 2013-02-21

**Authors:** A Pradhan, B McGuire, A Charles

**Affiliations:** 1 University of California, Los Angeles, USA

## Introduction

The mechanisms underlying the progression of migraine from an episodic to a chronic process are poorly understood, and the development of new therapies for chronic migraine has been slow, in part due to the lack of clinically relevant animal models.

## Objectives

The objectives of this study are to develop and characterize a new mouse model for chronic migraine, based on repetitive intermittent exposure to the human migraine trigger nitroglycerin [1,2]. A goal of this model is to test the effect of known and potential acute and preventive migraine therapies, in order to gain understanding regarding mechanisms of action and to develop preclinical evidence to support use of new treatments in patients with chronic migraine.

## Methods

Nitroglycerin was administered IP to mice every second day for 9 days. Basal and nitroglycerin-evoked mechanical hypersensitivity was evaluated using manual von Frey hair stimulation of the hindpaw.

## Results

Acute nitroglycerin administration evoked mechanical hyperalgesia in a dose dependent manner. Chronic intermittent treatment with nitroglycerin induced a progressive and sustained basal (T2) after rTMS. We measured changes in 1st block amplitudes and in habituation slopes for N1P1 and P1N2 components as outcome measures. Results in the TBE group (n=13), 1st block P1N2 amplitude and habituation of N1P1 increased after the stimulation, with partial recovery at 3 hours. Interestingly, habituation of P1N2 increased with time after the stimulation and was more pronounced at 3 hours compared to baseline or immediately after rTMS. In the QPI group (n=11), we found a post-stimulation reduction of 1st block amplitude, which also increased with time and was greater at 3 hours than immediately after the stimulation. No significant effect was found for the TBI and QPE protocols.

## Conclusion

Excitatory theta burst and inhibitory quadripulse rTMS are thus able to modify durably and differentially cortical activation level and VEP habituation. The former could be potentially useful in the preventive treatment of episodic migraine while the latter could have a beneficial effect in chronic migraine.
